# Vocal Tract Resonance Detection at Low Frequencies: Improving Physical and Transducer Configurations

**DOI:** 10.3390/s23020939

**Published:** 2023-01-13

**Authors:** Jithin Thilakan, Balamurali B.T., Sarun P.M., Jer-Ming Chen

**Affiliations:** 1 Erich Thienhaus Institute, Detmold University of Music, 32756 Detmold, Germany; 2 Department of Science, Mathematics, and Technology, Singapore University of Technology and Design (SUTD), Singapore 487372, Singapore; 3 Department of Physics, Indian Institute of Technology, Indian School of Mines, Dhanbad 826004, Jharkhand, India

**Keywords:** vocal tract resonance, acoustic impedance, acoustic transducer

## Abstract

Broadband excitation introduced at the speaker’s lips and the evaluation of its corresponding relative acoustic impedance spectrum allow for fast, accurate and non-invasive estimations of vocal tract resonances during speech and singing. However, due to radiation impedance interactions at the lips at low frequencies, it is challenging to make reliable measurements of resonances lower than 500 Hz due to poor signal to noise ratios, limiting investigations of the first vocal tract resonance using such a method. In this paper, various physical configurations which may optimize the acoustic coupling between transducers and the vocal tract are investigated and the practical arrangement which yields the optimal vocal tract resonance detection sensitivity at low frequencies is identified. To support the investigation, two quantitative analysis methods are proposed to facilitate comparison of the sensitivity and quality of resonances identified. Accordingly, the optimal configuration identified has better acoustic coupling and low-frequency response compared with existing arrangements and is shown to reliably detect resonances down to 350 Hz (and possibly lower), thereby allowing the first resonance of a wide range of vowel articulations to be estimated with confidence.

## 1. Introduction

Tracking vocal tract resonance reliably during both speech and singing is important in voice research. One such non-invasive and reliable technique [1,2,3,4,5,6,7,8,9,10] is to introduce a broadband excitation signal (acoustic current) just outside the speaker’s mouth during phonation, and the resulting acoustic pressure (arising from acoustic interactions with the vocal tract, lip aperture and radiation load of the face) is recorded and processed to reveal vocal tract resonances with a resolution in the order of 10 Hz. Due to its direct, non-invasive and ecological approach, this method complements other methods of investigating vocal tract articulation such as video fluoroscopy, electromagnetic articulography (EMA) and magnetic resonance imaging (MRI) by allowing researchers direct access to acoustic information during phonation.

During speech and singing, the acoustic response of the vocal tract during phonation can be modeled simply as a baffled acoustic duct, effectively closed at one end (the glottis is adducted during phonation; so we may consider the tract to be closed for all frequencies except for the phonation frequency and its harmonics) and open at the lips. Now, the subject’s face acts as a baffle and reduces the solid angle available for acoustic radiation (see Appendix A); so considering the open lips as a point source, the radiation impedance *Z_Rad_* seen at the lips is

*Z_Rad_* = *αz*
*jkr*/(1 + *jkr*) (1)

where *j* is the imaginary unit number, *r* is the radial distance from the source, *k* is the wavenumber given by *2πf/c*, *f* is the frequency, *c* is the speed of sound, *α* is a geometrical structural factor, and *z* is the specific impedance of the air [1]. On the other hand, the vocal tract has varying internal geometry depending on the articulation required. Accordingly, we can make an approximation of the vocal tract as an open-closed pipe with acoustic impedance *Z_Pipe_*,

*Z_Pipe_ = Z_0_* ((1 + *j* tanh(*αL*) tan(*ωL/v*))/(tanh(*αL*) *+ j* tan(*ωL/v*))) (2)

where *L* is the length of the pipe, *ω* is the angular frequency, *v* is the phase velocity, *Z_0_* is the characteristic acoustic impedance given by *ρc/S*, in which *ρ* is the density of the medium (air), *c* the speed of sound, and *S* is the cross-sectional area [11]. Vocal tract resonances (seen at the open lips) are indicated as local minima in the *Z_Pipe_* spectrum. Accordingly, the resulting combination of *Z_Pipe_* and *Z_Rad_* is seen at the lips acts in parallel [1]; so

*Z_||_* = 1/(1/*Z_Pipe_* + 1/*Z_Rad_*). (3)

**Figure 1:**
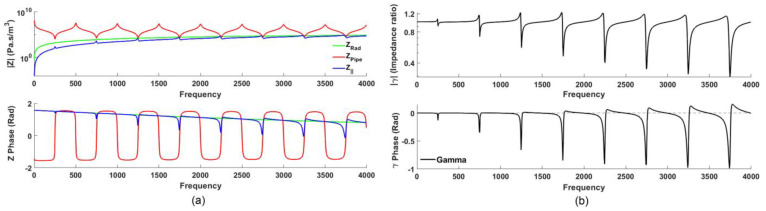
(**a**) Impedance (analytical model) for a theoretical baffled open-closed cylinder with a length of 340 mm and a radius of 13 mm: magnitude (upper), and phase (lower) spectra plotted for open-close *Z_Pipe_* (red), *Z_Rad_* (green) and *Z_||_* (blue); (**b**) *γ* (ratio of *Z_||_* to *Z_Rad_*) spectrum (black), magnitude (upper) and phase (lower). The “notches” (magnitude) and “dips” (phase) indicate resonances in *Z_Pipe_* [9].

Since *Z_Rad_* varies monotonically with frequency (it is resonance-free), while *Z_Pipe_* exhibits local maxima and minima (anti-resonances, and resonances, respectively), the resulting *Z_||_* spectrum accordingly indicates resonances as narrow inverted-V notches (see Figure 1a, *|Z|* plot). However, because *Z_Pipe_, Z_Rad_* and *Z_||_* vary dramatically over several orders of magnitude, it is advantageous to ‘normalize’ *Z_||_* and thus improve the signal to noise ratio and visual acuity to better indicate the presence of vocal tract resonances by dividing *Z_Rad_* to yield the dimensionless quantity *gamma* (Figure 1b),

*γ* = *Z_||_/Z_Rad_*. (4)

Figure 1 demonstrates the analytical relationship among *Z_Rad_*, *Z_Pipe_*, *Z_||_* and the resulting *γ* spectrum, for a theoretical baffled open-closed cylinder with a length of 340 mm and a radius of 13 mm, showing resonances ~250, 750, 1250, 1750, 2250, 2750, 3250, 3750 Hz (ignoring end effects). At these frequencies, pipe resonances (*Z_Pipe_* minima) are revealed to be bounded between the local maxima and minima (a distinctive “overshooting sigmoid-like” feature) associated with a steep local negative slope (‘notch’, Figure 1b, top) in the resulting gamma *magnitude* spectrum. Additionally, in the gamma *phase* spectrum, these resonances are further reflected clearly as acute local minima (‘dips’) that deviate sharply from zero phase (Figure 1b, bottom). These two spectral features identified in the gamma magnitude and phase spectra, respectively, are equivalent despite being in different representations and thus offer two independent means of identifying vocal tract resonances and reliably estimating their frequencies.

It is worth noting that both the resulting parallel ‘notch’ and ‘dip’ features in gamma spectra are very weak at low frequencies because *Z_Rad_* is weak at low frequencies—an acoustic ‘short-circuit’ of sorts. Consequently, both ‘notch’ and ‘dip’ features are easily overwhelmed at low frequencies when there is a noticeable signal to noise ratio in the measurement (it couples poorly for resonances arising below ~500 Hz). Because of this, our study seeks to identify alternative physical arrangements which may improve resonance detection below 500 Hz by optimizing the acoustic interaction between the vocal tract (or other target cavities of interest) and the transducers used.

## 2. Materials and Methods

To make gamma measurements (after [1,2,3,4,5,6,7,8,9]), a quasi-broadband excitation signal consisting of harmonics (Δ*f* = 5.4 Hz) ranging between 100 and 4000 Hz is generated and played by a mini-loudspeaker (HP Bluetooth Mini 300), which supplies the acoustic current at the lips (here, we are focused on detecting resonance frequency, rather than absolute *Z*); the resulting acoustic pressure is detected using a small lavalier microphone (Audio Technica ATR3350), also located at the lips (because we are trying to estimate resonance frequencies from gamma, and are not interested in measuring absolute *Z*, the constant current condition is not crucial here; the mass and density of the mini-loudspeaker diaphragm are still much larger than the air it is moving). Both the loudspeaker and the microphone are connected to a laptop computer via a USB DAC (SoundBlaster Play! 3 from Creative technologies, Singapore).

Prior to measurement, the excitation signal is first calibrated using the *Z_Rad_* associated with the flange (approximating the subject’s face) such that acoustic energy is distributed smoothly over the frequency range of interest; this calibration step also takes into account the acoustic effects associated with the physical presence of the loudspeaker and the microphone configured at the cylinder opening (approximating the lips) [2,9], and also the frequency response of the loudspeaker itself. Next, the calibrated signal is introduced while the subject phonated various target vowels and the resulting pressure signal is collected to compute *Z_||_* and subsequently the gamma spectrum, yielding vocal tract resonance information.

**Figure 2:**
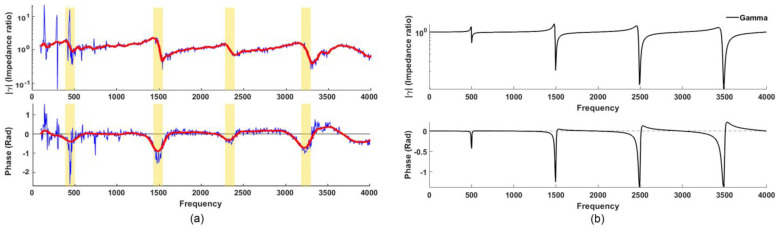
(**a**) Experimental *γ(f)* measured while phonating the neutral vowel /ə/ (in the target word “herd”), magnitude (above), and phase (below). Blue line shows the raw measurement, while the red line shows the smoothed with Savitsky–Golay algorithm [14] and interpolated spectra (having removed harmonics of the voice), revealing vocal tract resonances as “notches” (magnitude spectrum) and “dips” (phase spectrum), indicated by yellow vertical bands; (**b**) corresponding analytical *γ(f)* spectra modeled for an ideal baffled open-closed cylinder with a length of 170 mm; the “notches” and “dips” correspond closely in frequency and general structure with the experimental spectra in (**a**).

Figure 2a shows a typical *γ(f)* measurement (reported earlier in [9]) made while a speaker is phonating the neutral vowel /ə/ (as in the word “herd” [12,13]): the blue line shows the raw measurement (this includes harmonics of the phonating voice—a useful artifact that indicates the speaker’s phonatory *f_0_*) while the red line (smoothed and interpolated spectrum of the raw measurement) reveals four distinct resonances (“notches” and “dips” in the magnitude and phase spectra, respectively) at approximately 460, 1490, 2330 and 3210 Hz. Figure 2b, on the other hand, shows the analytically determined gamma for an ideal baffled open-closed cylinder with a length of 170 mm and a radius of 13 mm, resulting in resonances at ~500, 1500, 2500, and 3500 Hz (here we ignore end effects and assume a fully closed glottis). The resonances in Figure 2a,b (experimental vs. analytical) show good correspondence, especially the first two resonances (important for vowels). In practice, the open end of the cylinder will have an end correction (~0.6r) which lowers the resonance frequencies somewhat; however, during phonation, the glottis is slightly opened, which has the effect of raising the resonances [5]; therefore, these two effects happen to cancel out, resulting in resonances close to the theoretical closed-open pipe. (In this paper, we refer to the first, second, third, and fourth resonances as R1, R2, R3, and R4, respectively).

As highlighted above and consistent with Figure 1, because *Z_Pipe_* is in parallel with *Z_Rad_*, at low frequencies where *Z_Rad_* is weaker than *Z_Pipe_* (our measurement target), the first vocal tract resonance easily becomes obscured when there is measurement noise. To address this issue, we now explore different physical configurations of the acoustic current source (mini-loudspeaker) and acoustic pressure sensor (microphone) arranged about the lips, such that we may identify the optimal configuration.

**Figure 3:**
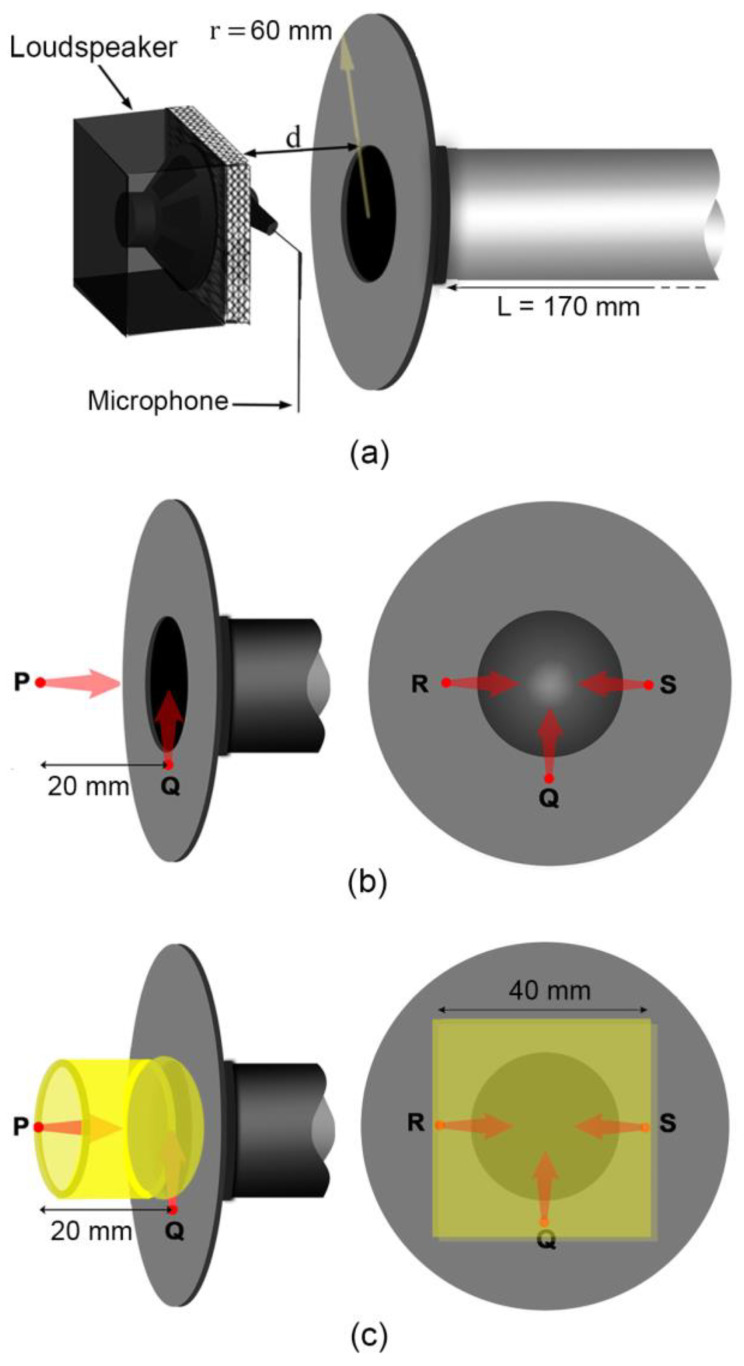
(**a**) Schematic showing default configuration of loudspeaker and microphone used to make gamma measurements (C1); (**b**) P, Q, R, and S indicate the locations and orientations of the loudspeaker and microphone in C1 to C5; (**c**) for C6 to C10, similar P, Q, R and S, but now with acoustic foam applied (indicated in yellow): C6, C7, and C8 utilize a hollow cylindrical foam (left) while C9 and C10 have a foam ‘cap’ filling in the space between the transducers and flange (right).

For consistency, instead of a human subject, we use a physical model as an indicative measurement target (dimensions are approximated from [13]): to represent the phonating vocal tract with open lips, we employ a 170 mm cylindrical pipe of a uniform radius (*r*_0_ = 13 mm), terminated with an almost-closed 3 mm aperture approximating an open glottis (associated with phonation) at the far end, along with a circular flange (radius 60 mm, representing the face, which constrains and reduces the solid angle available for acoustic radiation emanating from the lips [15]) at the open end (“lips”) as shown in Figure 3a.

**Figure 7:**
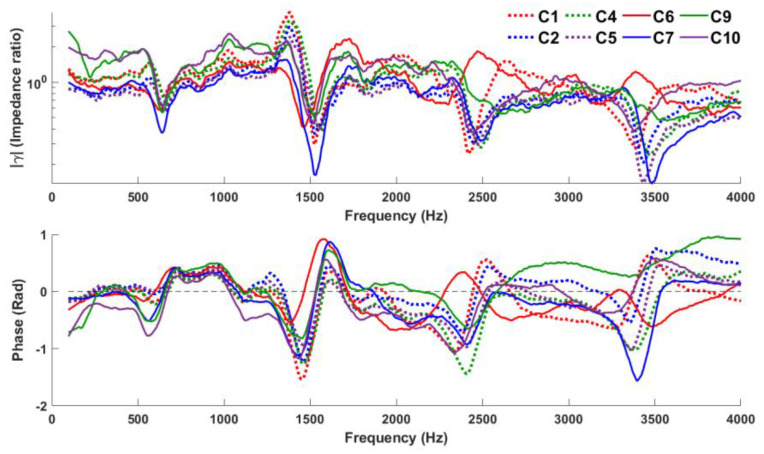
Comparison of eight configurations (C1 to C10, excluding C3 and C8).

Various configurations of loudspeaker (source) and microphone (sensors) at location and orientations P, Q, R, and S with respect to the open lips (see Figure 3) are applied to the physical vocal tract model, including the presence of acoustic foam filling in the space between point P and the flange. P is co-axial with the pipe and looks directly into the open pipe, at a separation *d* = 20 mm from the flange; Q, R and S are located on the flange, orthogonally oriented to each other and face radially inwards. (Because we are interested in low-frequency resonances < 500 Hz, the distances separating P, Q, R, and S are relatively smaller than the characteristic wavelength at these frequencies, and so the source and sensor can be considered to be in phase.) Table 1 describes the 10 configurations explored in this study (numbered henceforth as “C1”, “C2”, etc.), where C1 to C5 are without acoustic foam and C6 to C10 include acoustic foam applied “at the lips”. The acoustic foam with a density of ~26 kg.m^−3^ was introduced as a hollow cylinder with a length of 2 cm in C6 to C8, whereas it was used to fill the space between the transducers and the flange (~4 cm × 4 cm × 2 cm) in C9 and C10 (further acoustic properties of foam can be found at [16]). Open-celled acoustic foam can influence the measurement response of the system [17] and configuration by improving the acoustic coupling between the transducers and waveguide cavity at low frequencies by raising the *Z_Rad_* at low frequencies. (While it may be expected that the presence of the foam near the open radiating end of the pipe may introduce end effects influencing the resonance frequencies, the experimental results in Figure 7 demonstrate otherwise and this requires further analysis).

The experimental setup is housed in an anechoic chamber, mounted rigidly on two stands at a height of 1 meter to minimize reflections from the floor (see Appendix B), and reflections from the stands are assumed to be negligible. For each configuration, four measurements were made, and this involved ‘setting up’ and ‘tearing down’ each time to make a fresh measurement in order to ensure good measurement ‘typicity’ was achieved.

## 3. Results and Discussion

### 3.1. Examining the Validity of the Setup

**Figure 4:**
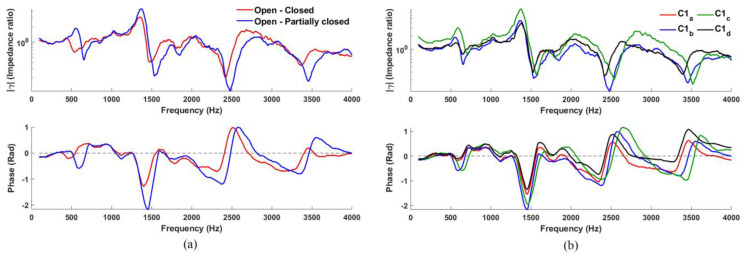
(**a**) Measurement of configuration 1 for “glottis” completely and partially closed; (**b**) four typical *γ(f)* measurements made on a 170 mm pipe using configuration 1, magnitude above and phase below; resonances are indicated in both magnitude and phase plots at approximately 590, 1450, 2370, and 3350 Hz with less than 2% disagreement.

Figure 4a shows the *γ* output (both amplitude and phase) corresponding to configuration C1 for an ‘open-fully closed’ pipe with a length of 170 mm (red line), and that of an ‘open-partially closed’ pipe that better resembles the real glottis in phonation (blue line). The open-fully closed pipe indicates resonances R1, R2, and R3 at 475, 1420, and 2360 Hz, respectively (within ~5% error compared to the 500, 1500, 2500 Hz expected; R4 cannot be easily estimated) thus confirming the validity of the experimental setup. The open-partially closed pipe simulating phonation with a slightly open glottis resulted in resonances at 595, 1455, and 2390 Hz, with R1 now slightly raised as expected, due to the presence of the partially closed glottis [5].

### 3.2. Analyzing Various Configurations

#### 3.2.1. Configuration 1

Four typical *γ(f)* for C1 are shown in Figure 4b for both magnitude and phase. These measurements (each made after tearing down and setting up with a fresh calibration) show good repeatability and agreement across all four plots: the resonance frequencies are at approximately 590, 1450, 2370, and 3300 Hz for both magnitude and phase plots (“notches” and “dips”, respectively), with less than 2% disagreement in general.

#### 3.2.2. Configuration 2, 3, 4, and 5

**Figure 5:**
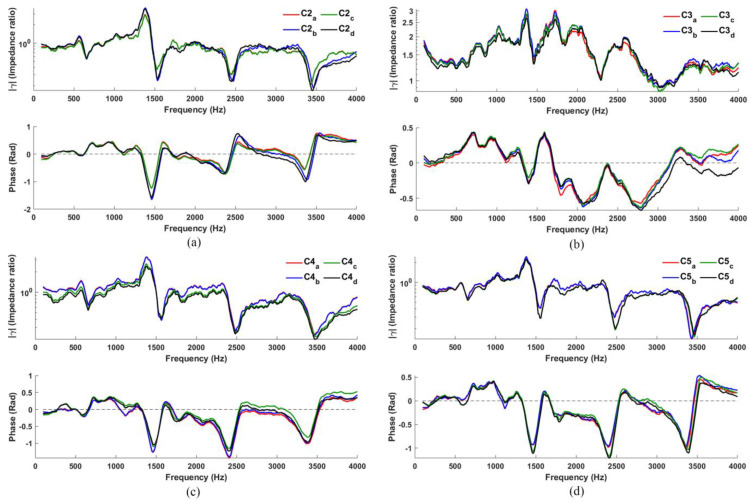
Typical *γ(f)* measured using configuration: (**a**) C2; (**b**) C3; (**c**) C4; (**d**) C5.

Figure 5 shows the resulting *γ(f)* for C2, C3, C4, and C5. In general, C2, C4, and C5 do not perform poorer than C1; in fact, it may be argued that R3 and R4 (high-frequency resonances) are even more distinguished here than in C1 for both magnitude and phase spectra.

In contrast, C3 performs rather poorly (in both magnitude and phase), but with some effort, R2 and R3 may still be alluded (but only in the magnitude spectra). The weaker performance here may be attributed to poor coupling between the loudspeaker, microphone and the cavity: (1) the loudspeaker and microphone have a direct path that minimizes contributions from the cavity; (2) the loudspeaker is now placed against the flange, thereby increasing reflections of the loudspeaker signal received at the microphone at position P, and thus together compromise the overall signal received. This contrast with the other configurations: the loudspeaker in C2 radiates directly into the cavity. Hence, its reflections (out of the cavity) are easily detected by the microphone kept at the flange by the cavity; C4 and C5 both have loudspeaker-microphone paths that necessarily interact with the cavity, accounting for improved resonance responses in both cases. Since this investigation is about identifying the optimal configuration for detecting vocal tract resonances and estimating its frequencies, C3 (and its corresponding configuration, C8) will not be considered for further analysis.

Considering C2, C4, and C5, the upper resonances (R3 and R4) in C4 and C5 are rather more pronounced in the phase spectra, enabling easier resonance detection and allowing its frequency to be better estimated. However, R1 is slightly more pronounced in C4.

#### 3.2.3. Configuration 6, 7, 9, 10

**Figure 6:**
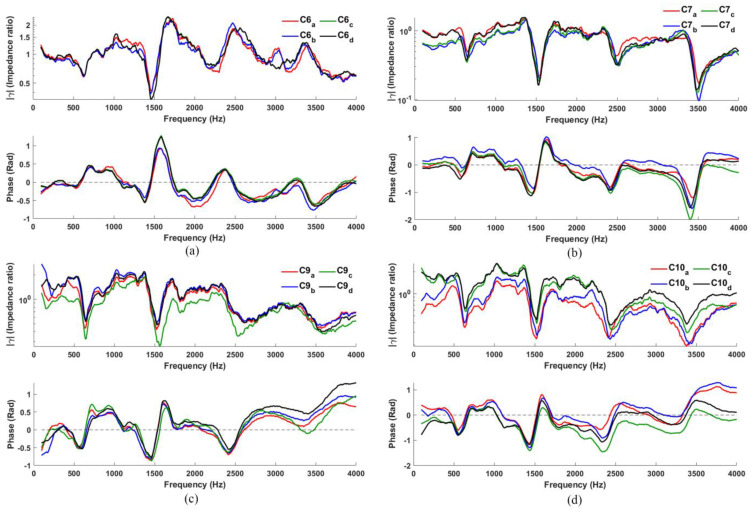
Typical *γ(f)* measured using configuration: (**a**) C6; (**b**) C7; (**c**) C9; (**d**) C10.

Open-celled acoustic foam is introduced in the void space of C1, C2, C4, and C5 to obtain C6, C7, C9, and C10 (see Table 1); corresponding measurements are shown in Figure 6. Figure 6a shows the results for C6. The magnitude plot shows R2 clearly, with R1 weakly indicated, while R3 and R4 cannot be easily estimated; the dips in the phase plot are poorly defined. Since the foam separates the microphone-loudspeaker setup from the flange in C6, (1) the signal arriving from the loudspeaker is reduced at the cavity, and (2) the interaction of the microphone with the signal reflecting from the cavity becomes weaker, together result in poor coupling in C6, as compared with C1. In contrast, with foam included, the low-frequency responses of C7, C9 and C10 (Figure 6b–d) instead seem to be improved when compared to their earlier versions C2, C4, and C5.

Figure 7 compares the performance of eight configurations (C1 to C10, excluding C3 and C8). To make this comparison, we select the ‘median’ measurement in each configuration (i.e., avoiding curves with extreme variations), which we consider to be a typical measurement. C3 and C8 are excluded for the aforementioned reasons.

Acoustic foam is somewhat acoustically transparent and denser than air; as a porous material, its characteristic impedance will be higher than air [17]. So, when the acoustic foam is introduced (C7, C9, and C10), the increased radiation impedance at the lips due to the foam results in an improved low-frequency response for these configurations compared to those without the foam (resonance frequencies estimated remains consistent). As a result, the ‘dips’ and ‘notches’ in both phase and magnitude plots become more pronounced, particularly at low frequencies, thus allowing R1 (and R2) to be clearly indicated here. (The consistency of the resonance frequency indicated by the ‘dips’ and ‘notches’ across various configurations is in agreement with magnitude and phase observations reported by [18]). On the other hand, since the foam also disperses and absorbs sound more efficiently at high frequencies, the high-frequency responses for C9 and C10 do get weaker, but not for C7 which has a hollow core without foam.

If high-frequency resonances are the main interests, C4 and C5 (which do not use foam) indicate resonances most clearly. However, since the goal of our study is to determine the configuration with good low-frequency response, C7, C9, and C10 should be used. To identify the best among them, an investigation was carried out using a quantitative analysis as described in the next section.

### 3.3. Quantitative Analysis

The resonance responses of configurations presented in Figure 7 are quantitatively compared against C1 (the original configuration) by estimating the respective “Phase Q-values” and the “normalized GMMR” (as a proxy for quantifying the sharpness of the resonance detected).

#### 3.3.1. Phase Q-Value Analysis (Phase Spectra)

**Figure 8:**
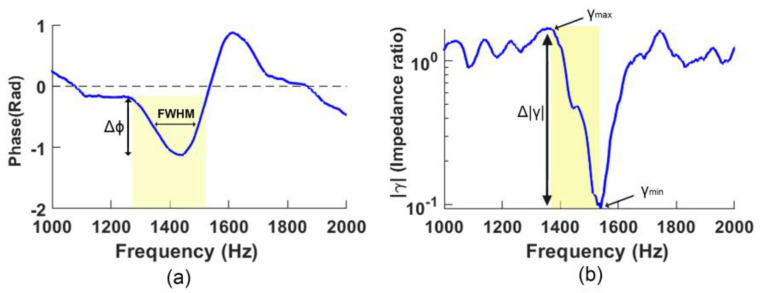
Example of quantifying a resonance based on the R2 from measurement C7 in Figure 7: (**a**) PQV = Δ*ϕ* /FWHM; (**b**) GMMR = *^γ^* _Local Maximum/_*^γ^* _Local Minimum_.

In this study, we defined the Phase Q-value (abbreviated as PQV) of the resonances indicated in the phase spectra (N.B.: not the traditional “Q-factor”) as a ratio of the depth of the local minimum (Δ*ϕ*, ‘dip’ with respect to the ‘knee’ left of the minimum) to the Full Width Half Maximum (FWHM) and can be used to quantify the severity of the dip (Figure 8a). The FWHM refers to the bandwidth in kHz between two points at half the depth of the local minimum (Δ*ϕ*), i.e.,

Phase Q-value (PQV) = Δ*ϕ*/ FWHM. (5)

Thus, as the PQV increases, the steepness and narrowness of the dip increases, and thereby indicates a resonance is strongly detected.

Table 2 presents PQVs of the first four resonances estimated manually for all configurations except C3 and C8, for the spectra presented in Figure 7. In general, configurations 4, 5, 7, 9, and 10 outperform C1. PQVs extracted for R1, C9 and C10 appear to have better low-frequency response than other configurations in general. R2 and R3 are visually prominent, and so the corresponding PQVs tend to range from moderate to high. Unlike their corresponding C4 and C5 analogs, C9 and C10 have much weaker R4. As pointed out above, this is expected because of the foam. Conversely, C7 which has a hollow foam core performs better than C2. (As mentioned above, R3 and R4 in C6 are not clearly indicated, and thus their PQVs are not defined in Table 2.)

#### 3.3.2. GMMR Analysis (Magnitude Spectra)

As seen above, the presence of resonance in the magnitude spectra is indicated with a distinctive “overshooting sigmoid-like” feature bounded between local maxima and minima associated with the negative slope centered about the resonance frequency (e.g., Figure 2 and Figure 8). The severity of the overshooting sigmoid helps us to be more confident that resonance is correctly identified and located. Therefore, in Figure 8b, we define

*^γ^* Maximum-Minimum Ratio (GMMR) = *^γ^* _Local Maximum/_*^γ^* _Local Minimum_ (6)

where a large GMMR denotes a clear identification of a resonance. (N.B. |*γ*| is plotted logarithmically.) This GMMR thus facilitates the comparison of resonances detected across measurements, as shown in Table 3 for configurations presented in Figure 7.

C9 and C10 show the largest GMMR for R1 (3.46 and 3.06, respectively), which indicate good low-frequency response, especially compared to the original configuration C1 (1.83), while C7 shows a moderate GMMR (2.58), the third best in the investigated configuration. This seems to reflect the benefit of the acoustic foam in C7-10; however, C6 seems like an exception. C4 performs the best for R3 (4.91); C5 performs the best for R4 (4.78), while C9 and C10 perform rather poorly (2.78 and 2.91 for R3, 1.87 and 2.01 for R4, respectively).

On the whole, C7, C9, and C10 perform the best, with C7 indicating resonances clearly overall. However, given that the objective of this paper is to better detect low-frequency resonances, it is worth noting C9 and C10. Consequently, C7, C9, and C10 are taken forward for further investigation along with the original configuration C1 in the next Section 3.4.

### 3.4. Study on Low-Frequency Resonances

**Figure 9:**
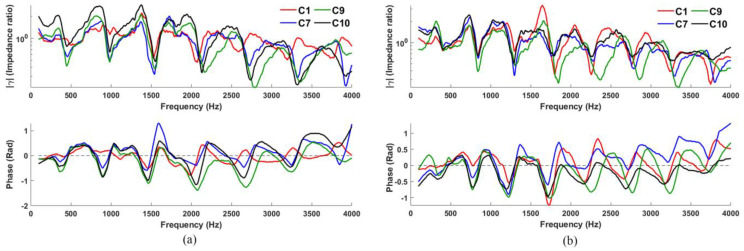
Comparison between C1, C7, C9, and C10 for a pipe length of (**a**) 283 and (**b**) 340 mm.

To identify the performance of the configurations for low frequencies, the gamma measurements corresponding to C1, C7, C9, and C10 (configurations resulted in the best performance in the previous analysis) are measured for pipes with lengths of 283 and 340 mm, with results shown in Figure 9 (for closed-open pipe, these lengths yield R1 at 300 and 250 Hz, respectively; the open glottis, however, will raise R1 somewhat). Among the four configurations explored, C9 and C10 in Figure 9 show the best low-frequency response (i.e., the first resonance can still be easily distinguished despite a longer “vocal tract”).

Table 4 presents the averaged PQV and GMMR for R1 estimated using C1, C7, C9, and C10 for four measurement repetitions as per Section 3.2.1. In most of the cases (comparing 170, 283, and 340 mm), the GMMR and PQV decrease with increasing pipe length. This is most clearly seen in C1, which struggles at 340 mm, with zero PQV but a modest GMMR. Nonetheless, C9 consistently yields the highest GMMR and PQV with only marginal loss in performance for every pipe length investigated, while C7 and C10 show intermediate performance with C10 presenting rather better than C7.

**Figure 10:**
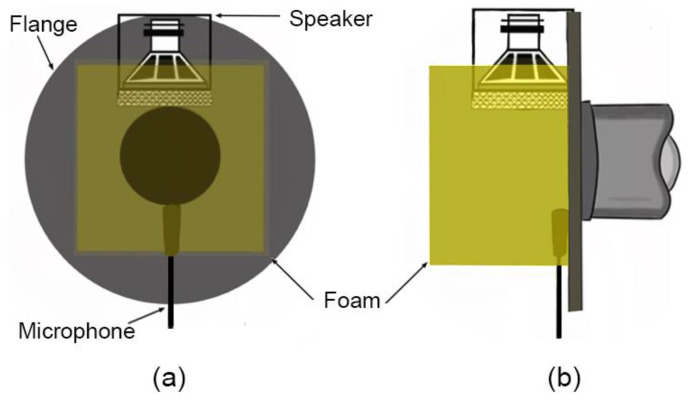
Schematic of configuration 9: (**a**) plane view; (**b**) side view.

Comparing resonances overall for extended systems, C9 consistently results in better GMMRs and PQVs compared to other configurations, due to better acoustic coupling that improves low-frequency response. Here, the source (loudspeaker) and sensor (microphone) are radially opposed across the pipe opening against the flange (Figure 10), such that the acoustic path between them (together with the foam) necessarily maximizes acoustic coupling with the pipe and flange compared with the other configurations explored in this study.

## 4. Conclusions

Building on earlier work using *γ(f)* to detect vocal tract resonance, we compared new physical configurations to identify the best configuration with better acoustic coupling and improved low-frequency responses. In addition to a qualitative analysis of *γ(f)*, two quantities are proposed (GMMR and PQV) to help quantify the relative performance of detecting resonances both from the magnitude and phase spectra of *γ*, respectively.

We found that the presence of acoustic foam positioned about the physical model (representing the mouth opening) raises the radiation impedance “at the lips”, resulting in an improved low-frequency response compared to configurations without foam. Further, we identified that configuration 9 (C9) (where the source (loudspeaker) and sensor (microphone) are radially opposed across the pipe opening and are kept close to the flange, coupled with acoustic foam filling the void between them) gives the best results in terms of acoustic coupling and low-frequency response among all other configurations explored. We surmise that since the microphone and loudspeaker here are kept close to the flange and are radially opposed, the acoustic path shared across them necessarily interacts with the cavity (vocal tract) located between them, while also reducing the direct acoustic interaction between them by increasing the acoustic path.

Together, the resonances detected in configuration 9 are more pronounced, particularly at low frequencies, thus allowing the first vocal tract resonance to be clearly indicated at least down to 350 Hz. This will allow the first resonance of a wide range of vowel articulations to be estimated with confidence.

## Figures and Tables

**Table 1:** Description of the configuration setup.

Configuration	Location of Microphone and the Loudspeaker	Configuration	Location of Microphone and the Loudspeaker
C1	Loudspeaker and Microphone “co-located” at P.	C6	C1 with a hollow cylindrical foam
C2	Loudspeaker at P, Microphone at Q.	C7	C2 with a hollow cylindrical foam
C3	Loudspeaker at Q, Microphone at P.	C8	C3 with a hollow cylindrical foam
C4	Loudspeaker at R, Microphone at S.	C9	C4 with foam filling in the space between transducers and flange
C5	Loudspeaker at R, Microphone at Q.	C10	C5 with foam filling in the space between transducers and flange

**Table 2:** PQV for configurations presented in Figure 7 (170 mm pipe).

	R1	R2	R3	R4
C1	1.57	10.60	4.24	4.53
C2	1.58	10.66	3.61	5.29
C4	1.81	7.56	5.96	4.56
C5	1.67	8.09	6.10	5.16
C6	1.10	4.78	N.A. ^1^	N.A. ^1^
C7	2.76	6.88	4.80	9.50
C9	4.55	4.54	3.22	0.83
C10	4.40	7.23	3.50	1.08

^1^ N.A. indicates no clear resonance was found.

**Table 3:** GMMR for configurations presented in Figure 7 (170 mm pipe).

	R1	R2	R3	R4
C1	1.83	13.25	3.47	3.15
C2	1.95	8.12	2.69	3.48
C4	2.10	8.57	4.91	3.54
C5	1.70	6.04	3.31	4.78
C6	1.50	3.18	N.A. ^1^	N.A. ^1^
C7	2.58	9.65	3.40	6.55
C9	**3.46**	4.18	2.78	1.87
C10	3.06	5.16	2.91	2.01

^1^ N.A. indicates no clear resonance was found.

**Table 4:** Averaged PQV and GMMR of the first resonance for configurations 1, 7, 9, and 10 for pipes with lengths of 283 and 340 mm.

Configuration	PQV		GMMR	
	283 mm	340 mm	283 mm	340 mm
C1	1.09	N.A. ^1^	1.29	1.36
C7	2.68	2.25	1.90	1.84
C9	**5.04**	**3.86**	**3.20**	**2.32**
C10	3.63	2.26	2.64	2.01

^1^ N.A. indicates no clear resonance was found.

**Figure A1:**
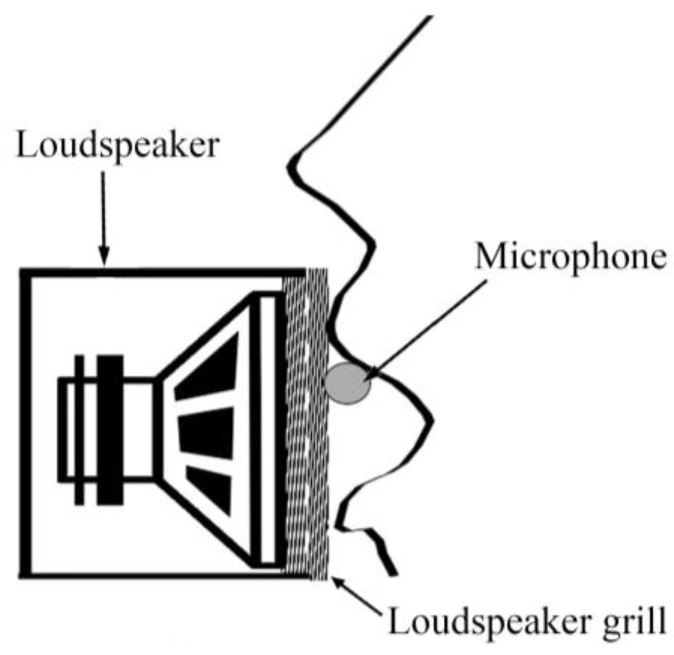
Schematic showing the microphone and loudspeaker configuration during a human subject measurement.

**Figure A2:**
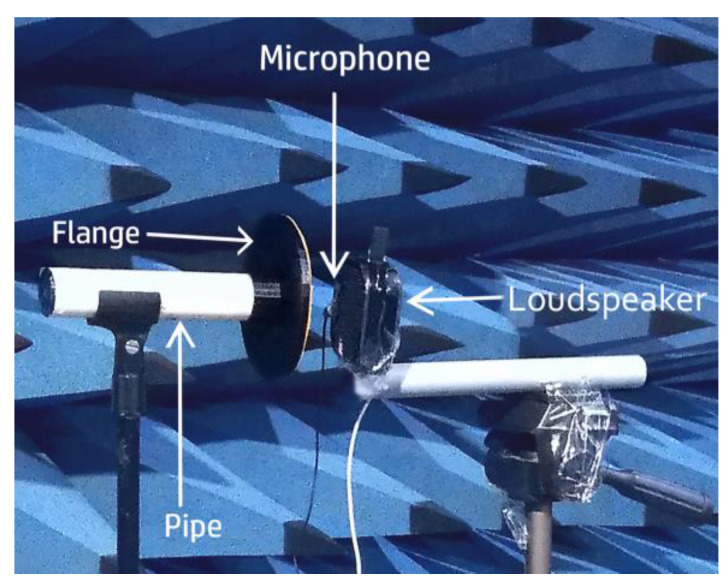
Schematic of the experimental setup.

## Data Availability

Not applicable.

## Appendix A

The schematic of the microphone and loudspeaker configuration of ACUZ-Lite during vowel measurement is shown in Figure A1 (from [9]). The phonating human subject is on the right side of Figure A1; the of the human subject is located at the microphone, the lower lips touches the grill of the loudspeaker, and the loudspeaker radiates directly into the open lips.

## Appendix B

The schematic of the experimental setup in the anechoic chamber is shown in Figure A2. The acoustic transducers, and the flange and pipe system were mounted rigidly on two stands at a height of 1 meter to minimize reflections from the floor.
